# CRIg on liver macrophages clears pathobionts and protects against alcoholic liver disease

**DOI:** 10.1038/s41467-021-27385-3

**Published:** 2021-12-09

**Authors:** Yi Duan, Huikuan Chu, Katharina Brandl, Lu Jiang, Suling Zeng, Nairika Meshgin, Eleni Papachristoforou, Josepmaria Argemi, Beatriz G. Mendes, Yanhan Wang, Hua Su, Weizhong Sun, Cristina Llorente, Tim Hendrikx, Xiao Liu, Mojgan Hosseini, Tatiana Kisseleva, David A. Brenner, Ramon Bataller, Prakash Ramachandran, Michael Karin, Wenxian Fu, Bernd Schnabl

**Affiliations:** 1 Department of Medicine, University of California San Diego, La Jolla, CA USA; 2 Department of Medicine, VA San Diego Healthcare System, San Diego, CA USA; 3 Division of Gastroenterology, Union Hospital, Tongji Medical College, Huazhong University of Science and Technology, 1277 Jiefang Avenue, 430022 Wuhan, China; 4 Skaggs School of Pharmacy and Pharmaceutical Sciences, University of California San Diego, La Jolla, CA USA; 5 University of Edinburgh Centre for Inflammation Research, The Queen’s Medical Research Institute, Edinburgh BioQuarter, Edinburgh, UK; 6 Division of Gastroenterology, Hepatology and Nutrition, Department of Medicine, University of Pittsburgh Medical Center, Pittsburgh Liver Research Center, Pittsburgh, PA USA; 7 Hepatology Program, Centro de Investigacion Medica Aplicada (CIMA), Liver Unit, Clinica Universidad de Navarra (CUN), Instituto de Investigacion de Navarra (IdisNA), University of Navarra, Pamplona, Spain; 8 Laboratory of Gene Regulation and Signal Transduction, Departments of Pharmacology and Pathology, School of Medicine, University of California San Diego, La Jolla, CA USA; 9 Department of Surgery, University of California San Diego, La Jolla, CA USA; 10 Department of Pathology, University of California San Diego, La Jolla, CA USA; 11 Department of Pediatrics, University of California San Diego, La Jolla, CA USA; 12 Department of Cancer Immunology, Genentech, South San Francisco, CA USA

**Keywords:** Alcoholic liver disease, Translational research, Monocytes and macrophages

## Abstract

Complement receptor of immunoglobulin superfamily (CRIg) is expressed on liver macrophages and directly binds complement component C3b or Gram-positive bacteria to mediate phagocytosis. CRIg plays important roles in several immune-mediated diseases, but it is not clear how its pathogen recognition and phagocytic functions maintain homeostasis and prevent disease. We previously associated cytolysin-positive *Enterococcus faecalis* with severity of alcohol-related liver disease. Here, we demonstrate that CRIg is reduced in liver tissues from patients with alcohol-related liver disease. CRIg-deficient mice developed more severe ethanol-induced liver disease than wild-type mice; disease severity was reduced with loss of toll-like receptor 2. CRIg-deficient mice were less efficient than wild-type mice at clearing Gram-positive bacteria such as *Enterococcus faecalis* that had translocated from gut to liver. Administration of the soluble extracellular domain CRIg–Ig protein protected mice from ethanol-induced steatohepatitis. Our findings indicate that ethanol impairs hepatic clearance of translocated pathobionts, via decreased hepatic CRIg, which facilitates progression of liver disease.

## Abstract (web summary)

CRIg is expressed on liver macrophages and binds Gram-positive bacteria to mediate phagocytosis, but it is not clear how its phagocytic functions contribute to liver homeostasis or disease. Here the authors report that ethanol impairs hepatic clearance of translocated pathobionts, via decreased hepatic CRIg, which facilitates progression of alcoholic liver disease.

## Introduction

Alcohol-related liver disease is the most prevalent liver disease worldwide^1–3^ and is the leading cause of liver transplantation in the United States^4^. Within this disease spectrum, alcoholic hepatitis is a severe acute-on-chronic liver failure, which is associated with 90-day mortality rates of 20–50%^5–8^. Early liver transplantation is the only curative therapy, but is solely available at select centers, to a limited group of patients^9–11^.

Chronic alcohol consumption leads to intestinal microbial dysbiosis^12–14^, which contributes to the development and progression of alcohol-associated liver disease^15–18^. Gut dysbiosis induces gut barrier dysfunction and allows viable bacteria to translocate from the gut to the liver^19,20^. We recently found that the presence of cytolysin-positive (cytolytic) *Enterococcus faecalis* (*E. faecalis*) in the intestine correlated with the severity of liver disease and with mortality in patients with alcoholic hepatitis. Translocated *E. faecalis* contribute to liver disease by producing cytolysin that can directly cause hepatocyte death^17^.

Bacteria that breach the intestinal barrier translocate into the blood stream and enter the liver, which serves as a second firewall to prevent their further spread^21^. Kupffer cells (KC) are the largest population of macrophages in the liver, where they phagocytose bacteria that escaped from the gut and arrived via the portal vein^21^. Phagocytosis of pathogens depends on opsonins, including antibodies and complement components^22^. The complement receptor of immunoglobulin superfamily (V-set and immunoglobulin domain containing 4, VSIG4, also called CRIg) is expressed primarily by KC^23^. CRIg binds to complement component C3b, iC3b and C3c, and mediates the clearance of C3b-opsonized bacteria^23,24^. CRIg can also directly, and independent of complement, bind to lipoteichoic acid—one of the cell wall components of Gram-positive bacteria—to support hepatic capture of circulating bacteria^25^. Independent from its phagocytic function, CRIg is able to reverse inflammation in experimental models of arthritis through inhibition of the alternative complement pathway, and has immunomodulatory functions in a mouse model of autoimmune type 1 diabetes^26,27^. Here, we investigated whether absence of CRIg or administration of a soluble CRIg-Ig protein affects progression of ethanol-induced liver disease via alteration in hepatic clearance of pathobionts.

## Results

### Liver tissue from patients with alcohol-related liver disease has lower levels of CRIg

**Fig. 1:**
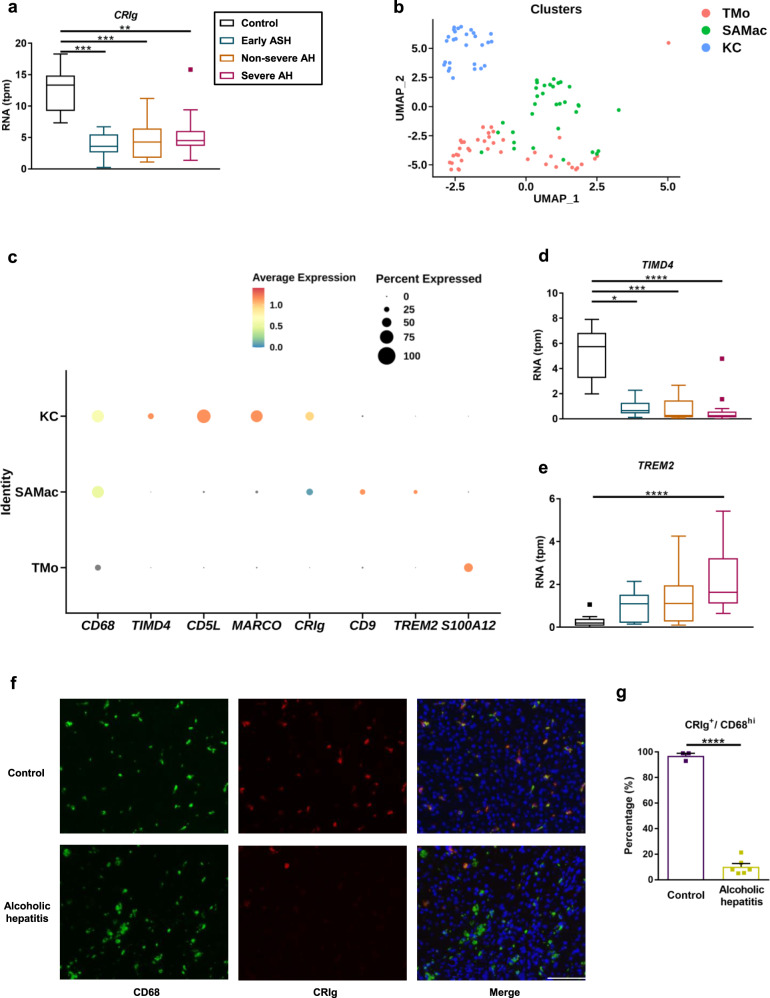
Patients with alcohol-related liver disease have less CRIg expression on liver macrophages. **a**, **d**, **e** RNA sequencing data of liver tissues from subjects without liver disease (Control; *n* = 10), patients with early alcoholic steatohepatitis (Early ASH; *n* = 12), non-severe alcoholic hepatitis (Non-severe AH; *n* = 11) and severe alcoholic hepatitis (Severe AH; *n* = 18). **b** and **c** Single-cell RNA sequencing data of liver tissues from subjects without liver disease (uninjured; *n* = 5) and alcohol-related cirrhotic patients (cirrhotic; *n* = 2). **a** mRNA level of *CRIg*. **b** UMAP annotating different macrophage cell types as KC (Kupffer cell), TMo (tissue monocyte) and SAMac (scar-associated macrophage). **c** Dot plot showing expression of *CRIg* and other stated marker genes in human liver macrophage subpopulations. **d** mRNA level of *TIMD4* (marker for Kupffer cells). **e** mRNA level of *TREM2* (marker for scar-associated macrophages). **f** Representative liver sections of CD68 and CRIg immunofluorescence staining. **g** Quantification of the stained liver sections. Scale bar = 100 μm. Results are expressed as median with interquartile range (**a**, **d**, **e**), or mean ± s.e.m. (**g**). *P* value is determined by two-sided Kruskal–Wallis test with Dunn’s post-hoc test (**a**, **d**, **e**), or two-sided Student *t*-test (**g**). *P < 0.05, **P < 0.01, ***P < 0.001, ****P < 0.0001.

To determine whether patients with alcohol-related liver disease have different hepatic CRIg expression levels, we analyzed RNA sequencing data of liver tissues from subjects without liver disease (controls) and patients with alcohol-related liver disease^28^. Compared with controls, liver tissues from patients with early alcohol-related steatohepatitis, severe and non-severe alcoholic hepatitis had a lower *CRIg* mRNA expression (Fig. 1a).

Recent single-cell RNA-seq (scRNAseq) studies of healthy and diseased human liver have defined heterogeneity in hepatic macrophages^29,30^, distinguishing transcriptionally distinct populations of tissue-resident KC, TMo, and scar-associated macrophages (SAMac) which expand in patients with chronic liver disease^30^. To assess which subpopulations of hepatic macrophages express *CRIg* in alcohol-related liver disease, we reanalyzed scRNAseq data from healthy and patients with alcohol-related liver disease^30^. Single cells were annotated as KC, TMo, and SAMac according to the original publication^30^ (Fig. 1b). *CRIg* is expressed primarily by KC (Fig. 1c), alongside other previously defined Kupffer cell markers including *TIMD4*, *CD5L* and *MARCO*^30^. In contrast, SAMac (distinguished by expression of *TREM2* and *CD9*) and TMo (distinguished by expression of *S100A12*) demonstrate lower expression of *CRIg* (Fig. 1c). Fewer KC were present in patients with alcohol-related cirrhosis (Fig. 1b and Supplementary Fig. 1a), whilst assessment of marker expression in whole liver tissue demonstrated that patients with alcohol-related liver disease also had reduced expression of other Kupffer cell marker genes (*TIMD4, CD5L* and *MARCO*) and increased expression of scar-associated macrophage marker genes (*TREM2* and *CD9*) (Fig. 1d, e and Supplementary Fig. 1b–d). Overall, these data indicate that altered hepatic macrophage composition with a reduction in KC contributes to the lower *CRIg* expression observed in alcohol-related liver disease.

Consistent with this, patients with alcoholic hepatitis showed a lower percentage of CRIg^+^/CD68^hi^ cells by immunofluorescence staining, indicating a reduced number of CRIg+ liver macrophages in patients with alcoholic hepatitis (Fig. 1f, g).

To determine regulation of CRIg expression on KC, primary mouse KC were treated with the TLR4 ligand LPS, heat-killed *E. faecalis* as TLR2 ligand, or inflammatory cytokines TNF and IL1B. All treatment groups showed reduced *CRIg* mRNA expression relative to control cells (Supplementary Fig. 1e). Taken together, our results indicate that reduced hepatic levels of CRIg in patients with alcohol-related liver disease is likely caused by a combination of inflammation-associated downregulation of CRIg and a reduced number of KC.

We also assessed T cell and neutrophil infiltration in liver tissues of patients with alcoholic hepatitis by H&E staining. Compared to control subjects, the sections of the six explanted livers showed advanced cirrhosis with thick fibrous bands containing mild lymphocytic inflammation and few neutrophils (Supplementary Fig. 1f). Occasional Mallory hyaline is noted which is related to oxidative injury and collapse of the cytoskeleton in the hepatocytes (Supplementary Fig. 1g).

### *CRIg*-deficient mice develop more severe ethanol-induced liver disease

We confirmed previous findings that CRIg is most highly expressed in liver in mice^23^—expression levels are low in other tissues (Supplementary Fig. 2a). Wild-type mice fed a chronic plus binge ethanol diet^31^ had significantly lower levels of hepatic *CRIg* than mice fed an isocaloric (control) diet (Supplementary Fig. 2b).

To study whether CRIg is involved in ethanol-induced liver disease, we fed *CRIg*-deficient mice and their wild-type littermates a chronic plus binge ethanol diet^31^. Compared with wild-type mice, *CRIg*^*−/−*^ mice developed more severe ethanol-induced liver injury, indicated by a significantly higher level of serum alanine aminotransferase (ALT) (Supplementary Fig. 2c) and increased hepatic steatosis (Supplementary Fig. 2d, e). *CRIg*^*−/−*^ mice fed the ethanol diet also had more liver inflammation, expressing significantly higher levels of mRNAs encoding inflammatory cytokines and chemokines (*Il1b*, *Cxcl1*, and *Cxcl2*) (Supplementary Fig. 2f).

To determine whether increased liver damage in *CRIg*^*−/−*^ mice was due to less-efficient bacterial clearance and possibly increased signaling from Gram-positive bacteria, we created double mutant-mice, with disruptions in the *CRIg* gene and toll-like receptor 2 gene (*Tlr2*). TLR2 is a pattern recognition receptor that binds components of Gram-positive bacteria and activates the inflammatory response, but does not function as phagocytosis receptor^32,33^. Compared with *CRIg*^*−/−*^ mice, mice with disruptions in both *CRIg* and *Tlr2* had less liver injury, steatosis, and inflammation—similar to ethanol-fed wild-type mice (Supplementary Fig. 2c–f). Disruption of *CRIg* or *Tlr2* did not affect intestinal absorption or hepatic metabolism of ethanol, based on serum levels of ethanol and hepatic levels of *Adh1* and *Cyp2e1* mRNAs, which encode the enzymes that metabolize ethanol in the liver (Supplementary Fig. 2g, h).

**Fig. 2:**
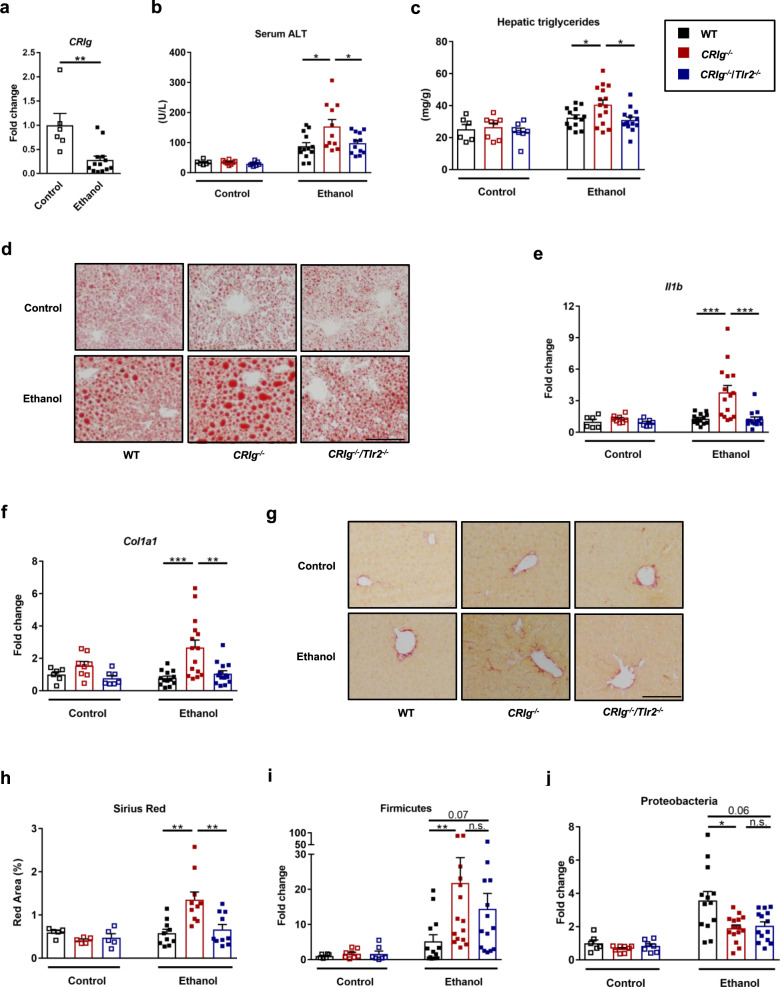
Deficiency of *CRIg* causes progression of ethanol-induced liver disease in mice. Wild-type (WT), *CRIg*^*−/−*^, and *CRIg*^*−/−*^/*Tlr2*^*−/−*^ mice were fed the Lieber DeCarli ethanol diet for 8 weeks. **a** Hepatic level of mRNA encoding *CRIg* in WT mice. **b** Serum levels of ALT. **c** Hepatic triglyceride content. **d** Representative oil red O-stained liver sections. **e** and **f** Hepatic levels of *Il1b* and *Col1a1* mRNAs. **g** Representative sirius red-stained liver sections. **h** Quantification of the sirius red-stained liver sections. **i** and **j** Hepatic DNA levels of Firmicutes and Proteobacteria, normalized to total amount of bacteria using universal 16S primers. Scale bar = 100 μm. Results are expressed as mean ± s.e.m. (**a**–**c**, **e**, **f**, **h**–**j**). *P* values among groups of mice fed with control diet or ethanol diet are determined by two-sided Student *t*-test (**a**) or one-way ANOVA with Tukey´s post-hoc test (**b**, **c**, **e**, f, **h**–**j**). *P < 0.05, **P < 0.01, ***P < 0.001.

We then studied the effects of CRIg loss in a model of more chronic liver disease, placing mice on an ethanol-containing Lieber DeCarli diet for 8 weeks^34^. Wild-type mice had reduced hepatic *CRIg* expression following ethanol feeding (Fig. 2a and Supplementary Fig. 3a, b). Ethanol-fed *CRIg*^*−/−*^ mice developed more severe liver injury, steatosis, and inflammation than wild-type mice; the increase in severity was not observed in *CRIg*^*−/−*^/*Tlr2*^*−/−*^ mice (Fig. 2b–e, Supplementary Fig. 3c, d). In addition, livers from ethanol-fed *CRIg*^*−/−*^ mice had increased expression of genes involved in fibrosis—namely collagen a1(I) (*Col1a1*) (Fig. 2f) and smooth muscle α-actin (*Acta2*) (Supplementary Fig. 3e), a marker of activated myofibroblasts and stellate cells, which contribute to the development of fibrosis. Livers from ethanol-fed *CRIg*^*−/−*^ mice also had elevated expression of transforming growth factor b1 (*Tgfb1*) (Supplementary Fig. 3f) and increased fibrosis staining (Fig. 2g, h). To assess whether *CRIg* deficiency affects liver bacteria, we determined the relative proportion of the Gram-positive phylum Firmicutes, and of the Gram-negative phyla Proteobacteria and Bacteriodetes in the liver. Compared with wild-type mice, *CRIg*^*−/−*^ mice had higher proportions of Gram-positive Firmicutes, but lower proportions of Gram-negative Proteobacteria (Fig. 2i, j) and Bacteriodetes (Supplementary Fig. 3g). Wild-type, *CRIg*^*−/−*^, and *CRIg*^*−/−*^/*Tlr2*^*−/−*^ mice had similar intestinal absorption and hepatic metabolism of ethanol (Supplementary Fig. 3h, i). We also assessed T cell and neutrophil infiltration in all groups of ethanol-fed mice and did not observe significant differences (Supplementary Fig. 4). Taken together, our results indicate that CRIg deficiency results in more severe ethanol-induced liver damage, and this exacerbation of disease is reduced in the absence of TLR2.

### CRIg protects from *E. faecalis*-exacerbated ethanol-induced liver disease

**Fig. 3:**
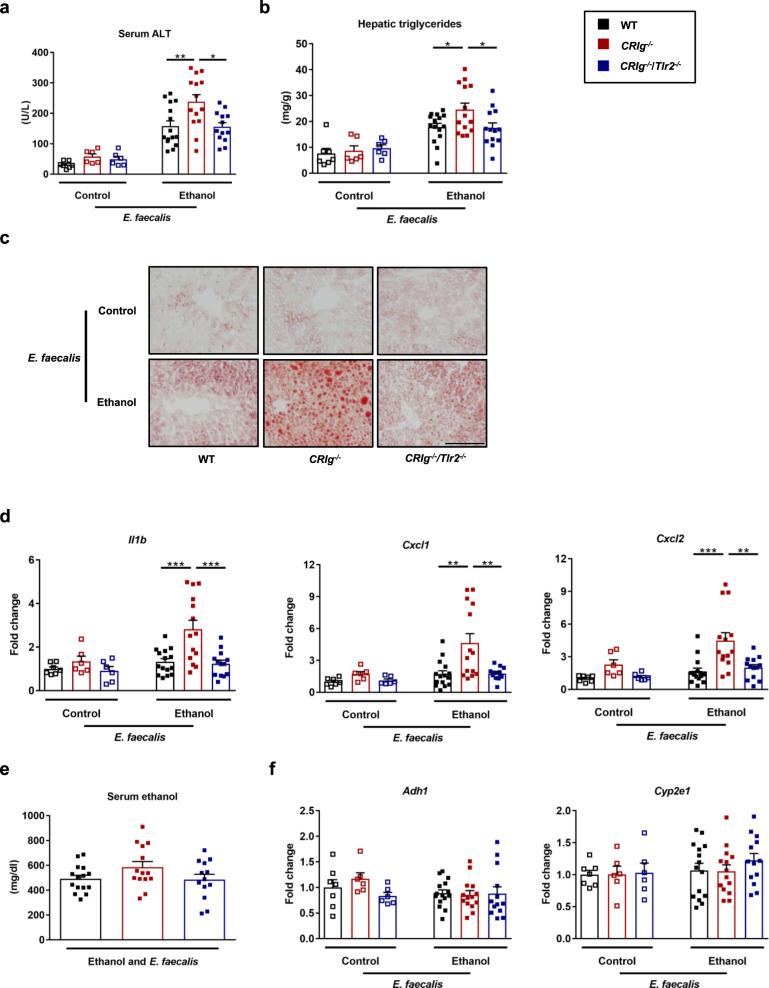
*CRIg*^*−/−*^ mice are more susceptible to *E. faecalis-* and ethanol-induced liver disease. WT, *CRIg*^*−/−*^ and *CRIg*^*−/−*^/*Tlr2*^*−/−*^ mice were placed on the chronic–binge ethanol diet and gavaged with a cytolytic *E. faecalis* strain (5 × 10^8^ colony forming units (CFUs)) every third day. **a** Serum levels of ALT. **b** Hepatic triglyceride content. **c** Representative oil red O-stained liver sections. **d** Hepatic levels of mRNAs encoding inflammatory cytokines and chemokines IL1B, CXCL1, and CXCL2. **e** Serum levels of ethanol in ethanol-fed mice. **f** Hepatic levels of *Adh1* and *Cyp2e1* mRNAs. Scale bar = 100 μm. Results are expressed as mean ± s.e.m. (**a**, **b**, **d**–**f**). *P* values among groups of mice fed with control diet or ethanol diet are determined by one-way ANOVA with Tukey´s post-hoc test (**a**, **b**, **d**–**f**). *P < 0.05, **P < 0.01, ***P < 0.001.

Oral administration of cytolytic *E. faecalis* promotes ethanol-induced liver disease in wild-type mice^17,35^. To determine whether *CRIg*^*−/−*^ mice are more susceptible to *E. faecalis*-exacerbated ethanol-induced liver disease, we gavaged mice with a cytolytic strain of *E. faecalis*^35^ and placed them on the chronic–binge ethanol diet. *CRIg*^*−/−*^ mice developed more severe liver injury, steatosis, and inflammation; this effect was reduced in *CRIg*^*−/−*^/*Tlr2*^*−/−*^ mice (Fig. 3a–d). *CRIg*^*−/−*^ mice also had more systemic inflammation as indicated by higher levels of circulating cytokines compared with their wild-type littermates and *CRIg*^*−/−*^/*Tlr2*^*−/−*^ mice (Supplementary Fig. 5a, b). No significant differences were observed in intestinal absorption and hepatic metabolism of ethanol among the three groups (Fig. 3e, f). These findings provide evidence that CRIg protects against *E. faecalis-*exacerbation of ethanol-induced liver damage.

### CRIg is required for capturing *E. faecalis* translocated from gut to the liver

**Fig. 4:**
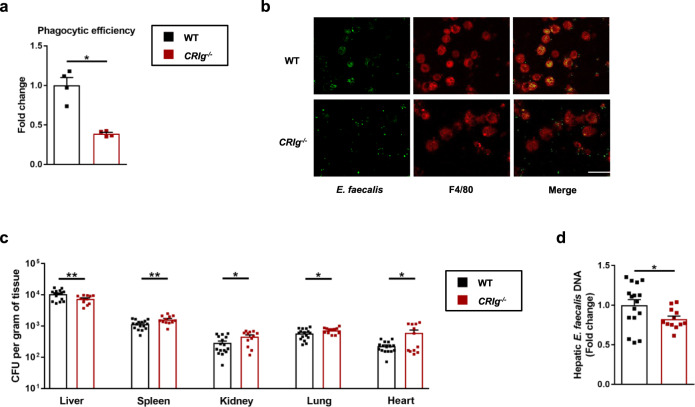
CRIg is required for capturing of *E. faecalis* translocated from gut to the liver. **a** and **b** *CRIg*^*−/*−^ mice and their WT littermates were placed on a chronic–binge ethanol diet and GFP-*E. faecalis* (2 × 10^7^ CFUs) was intravenously injected 1 h after ethanol-binge. Mice were sacrificed 1 h after injection and Kupffer cells were isolated. **a** Quantification of the stained cells. **b** Representative microscopy pictures. **c** and **d** *CRIg*^*−/*−^ mice and their WT littermates were fed the chronic-binge ethanol diet. One hour after the ethanol binge, mice were gavaged with *E. faecalis* (5 × 10^9^ CFUs). Bacterial load (**c**) and DNA levels (**d**) were determined 20 min after bacterial gavage. Scale bar = 10 μm. Results are expressed as mean ± s.e.m. (**a**, **c**, **d**). *P* values determined by two-sided Mann–Whitney test (**a**, **c**, **d**). *P < 0.05, **P < 0.01.

CRIg is important for capturing Gram-positive bacteria *Staphylococcus aureus* and *Listeria monocytogenes* that are circulating in the blood^23,25^. Therefore, ethanol-fed *CRIg*^*−/−*^ mice and their wild-type littermates were injected with fluorescently labeled *E. faecalis* and then KC were isolated. *CRIg*-deficient KC phagocytosed *E. faecalis* much less efficiently than KC isolated from wild-type mice (Fig. 4a, b), indicating that CRIg is important for phagocytosis of systemic *E. faecalis*.

To further characterize the phenotype of *CRIg*-deficient KC, primary KC were isolated from *CRIg*^*−/−*^ mice and their wild-type littermates, and stimulated with LPS. There was no difference in baseline or LPS-induced *Il1b*, *Tnf* and *Il10* mRNA expression, or in cell viability between wild-type and *CRIg*-deficient KC (Supplementary Fig. 6a–d).

To determine whether CRIg+ KC are also capable of capturing *E. faecalis* translocated from gut to the liver, we gavaged ethanol-fed *CRIg*^*−/−*^ mice and their wild-type littermates with fluorescently labeled *E. faecalis*. Due to deficiency in phagocytosis of KC, the number of translocated *E. faecalis* recovered from ethanol-fed *CRIg*^*−/−*^ mice were significantly lower in the liver but significantly higher in the spleen, kidney, lung, and heart shortly after ethanol binge (Fig. 4c, d). These data indicate that fewer translocated bacteria are captured in *CRIg*^*−/−*^ livers, resulting in increased numbers in the circulation and other organs. CRIg therefore captures *E. faecalis* that translocated from gut to liver.

### Extracellular domain of CRIg protects mice from ethanol-induced liver disease

**Fig. 5:**
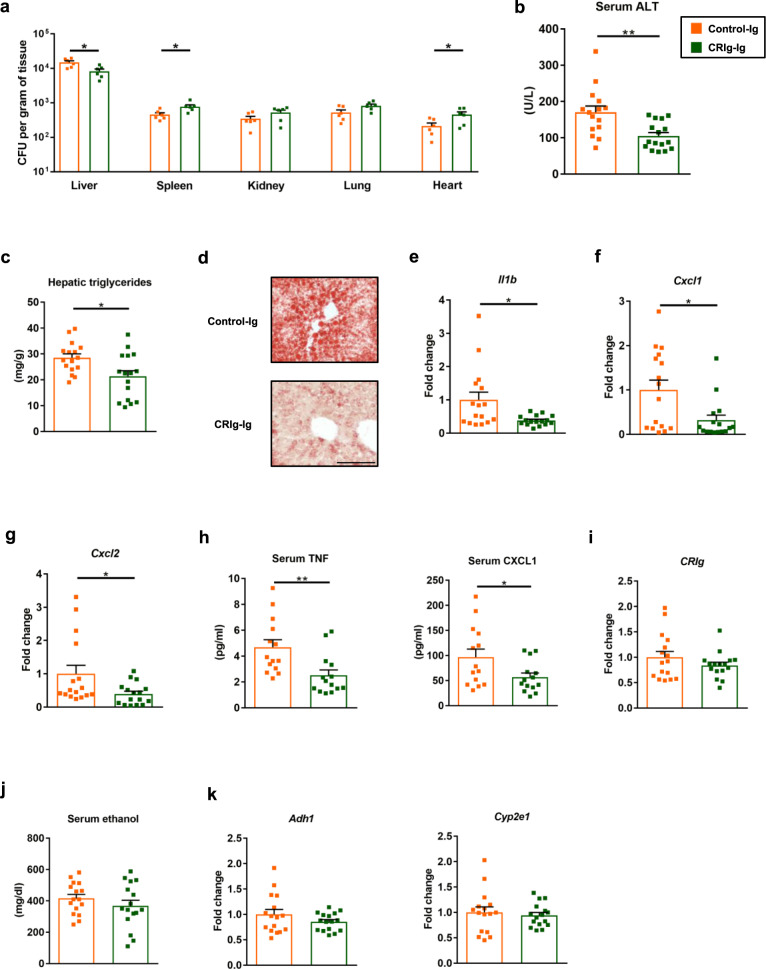
Extracellular region of CRIg protects mice from ethanol-induced liver disease. WT mice were fed the chronic–binge ethanol diet. **a** One hour after the ethanol binge, mice were given intravenous injections of control-Ig or CRIg-Ig (12 mg/kg body weight [BW]) and gavaged with *E. faecalis* (5 × 10^9^ CFUs). Bacterial load was determined 20 min after bacterial gavage. **b**–**k** One day before the ethanol binge, mice were given intravenous injections of control-Ig or CRIg-Ig (12 mg/kg body weight [BW]). **b** Serum levels of ALT. **c** Hepatic triglyceride content. **d** Representative oil red O-stained liver sections. **e**–**g** Hepatic levels of mRNAs encoding inflammatory cytokines and chemokines IL1B, CXCL1, and CXCL2. **h** Serum levels of inflammatory cytokines and chemokines TNF and CXCL1. **i** Hepatic level of mRNA encoding *CRIg*. **j** Serum levels of ethanol. **k** Hepatic levels of *Adh1* and *Cyp2e1* mRNAs. Scale bar = 100 μm. Results are expressed as mean ± s.e.m (**a**–**c**, **e**–**k**). *P* values determined by two-sided Student’s *t*-test (**a**–**c**, **e**–**k**). *P < 0.05, **P < 0.01.

muCRIg contains two regions—an extracellular region consisting of one immunoglobulin-like domain (CRIg-Ig) and an intracellular region with the transmembrane domain^23^. CRIg-Ig binds Gram-positive bacteria such as *S. aureus*, *L. monocytogenes*, *Bacillus cereus*, and *Enterococcus faecium*^25^. We confirmed that CRIg-Ig binds to Gram-positive *E. faecalis*, but not Gram-negative *E. coli* in vitro (Supplementary Fig. 7a). To determine whether CRIg-Ig is capable of binding to *E. faecalis* translocated from gut to the liver, ethanol-fed wild-type mice were injected with CRIg-Ig or control-Ig, and gavaged with fluorescently labeled *E. faecalis*. The number of translocated *E. faecalis* recovered from mice with CRIg-Ig were significantly lower in the liver but higher in other organs (Fig. 5a), indicating that translocated *E. faecalis* bound to CRIg-Ig escape Kupffer cell phagocytosis. We next determined whether *E. faecalis* bound to CRIg are recognized by TLR2. *E. faecalis* were incubated with CRIg-Ig or control-Ig, and used to stimulate primary mouse KC. *E. faecalis* incubated with control-Ig induced a stronger inflammatory response as compared with *E. faecalis* incubated and bound to CRIg-Ig (Supplementary Fig. 7b), indicating that *E. faecalis* binding to CRIg-Ig interferes with their ability to bind pattern recognition receptors such as TLR2.

We then investigated whether CRIg-Ig protects mice from ethanol-induced liver disease. Wild-type mice were fed a chronic-binge ethanol diet and then given intravenous injections of CRIg-Ig or control-Ig. Mice injected with CRIg-Ig developed less-severe liver injury (Fig. 5b), steatosis (Fig. 5c, d), hepatic inflammation (Fig. 5e–g) and systemic inflammation (Fig. 5h) than mice given the control-Ig but had no change in hepatic levels of CRIg or intestinal absorption or hepatic metabolism of ethanol (Fig. 5i–k). Together, our results indicate that CRIg protects against ethanol-related liver disease and might be developed as a therapeutic agent for alcohol-induced liver injury.

## Discussion

**Fig. 6:**
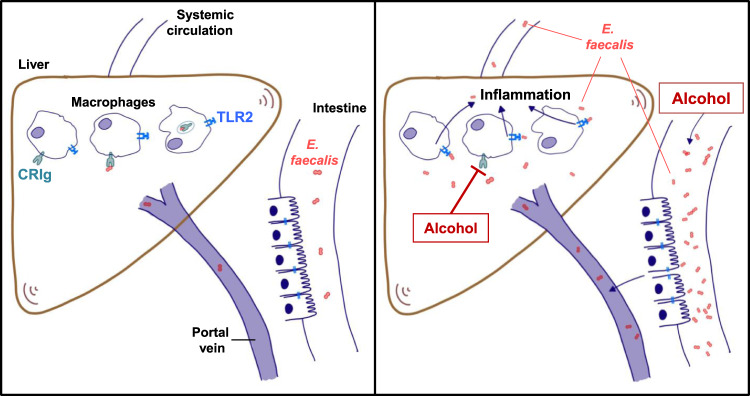
Alcohol reduces the clearance of pathobionts by Kupffer cells and contributes to liver disease. Left panel: During homeostasis, translocation of pathobionts from the gut to the liver is a rare event. Translocated *E. faecalis* are cleared and phagocytosed by CRIg on Kupffer cells. Right panel: Chronic alcohol consumption results in intestinal expansion of *E. faecalis*. These pathobionts escape the intestinal barrier and translocate to the liver. Alcohol changes hepatic macrophage composition and phenotype, and downregulates CRIg on Kupffer cells, thus contributing to reduced CRIg expression in the liver and reducing clearance of translocated *E. faecalis*. Hepatic and circulating *E. faecalis* are recognized by TLR2, a pattern recognition receptor that binds Gram-positive bacteria, resulting in hepatic inflammation and progression of alcoholic liver disease.

CRIg serves not only as a complement receptor, but is also important for pathogen recognition and clearance^23,25^. We recently showed that cytolysin-positive *E. faecalis* are increased in the intestine of patients with severe alcoholic hepatitis and their abundance correlates with mortality. These pathobionts escape the intestinal firewall^21^ and translocate to the liver, where they produce cytolysin, which damages hepatocytes^17^. In this study, we demonstrate that alcohol has detrimental effects on the liver as second firewall. Alcohol-related liver injury results in altered hepatic macrophage composition and phenotype, with a reduced number of CRIg+ KC ultimately attenuating the clearance of pathogenic bacteria. Mice deficient in CRIg developed more severe ethanol-induced liver disease than wild-type mice and had greater exacerbation of liver disease following oral administration of *E. faecalis* and ethanol feeding. Circulating *E. faecalis* are recognized by TLR2, a pattern recognition receptor that binds molecular patterns of Gram-positive bacteria, resulting in hepatic inflammation and chronic liver damage (Fig. 6). Ethanol therefore contributes to liver disease not only via its direct toxic effects on liver cells, but also by increasing intestinal passage of microbes into the blood stream and reducing liver CRIg expression, preventing clearance of bacteria from the liver.

We also showed that the effects of ethanol-mediated downregulation of CRIg on KC can be overcome by administration of soluble CRIg-Ig, which protects mice from ethanol-induced liver disease. As a complement receptor, CRIg modulates the immune system via different mechanisms. It binds to the activated complement component C3b and inhibits the alternative pathway of complement^24^. Soluble CRIg-Ig protein has been shown to have beneficial effects in models of autoimmune disease. The extracellular domain of CRIg prevents inflammation and bone loss in a mouse model of arthritis^26^. Administration of CRIg-Ig protein inhibits development of type I diabetes^36^, by attenuating proliferation of effector T cells and promoting differentiation and stability of T-regulatory cells^27,37^. CRIg helps maintain endogenous liver T- and natural killer T-cell tolerance to prevent concanavalin A-induced hepatitis^38^.

Little is known about the mechanisms by which CRIg modulates T-cell activity^39^. Although we cannot rule out that CRIg-Ig has beneficial T-cell modulatory effects, alcohol-associated liver disease is not considered to be mediated by a T-cell immune response. Furthermore, CRIg was shown to upregulate pyruvate dehydrogenase kinase-2 (PDK2) and reduce secretion of mitochondrial reactive oxygen species by macrophages, which inhibits their activation and inflammation in murine hepatitis virus strain-3 (MHV-3)-induced fulminant hepatitis^40^. We did not find a significant difference in expression of *Pdk2* among the groups of mice in all of our models, indicating that the exacerbation of ethanol-induced liver injury in *CRIg*^*−/−*^ mice was not due to a lower level of PDK2. In our model, CRIg-Ig acts as decoy receptor for Gram-positive pathobionts—it obscures their pathogen-associated molecular pattern and prevents activation of pathogen recognition receptors such as TLR2. Soluble CRIg-Ig protein might therefore be developed as a therapeutic agent for patients with alcoholic hepatitis, which has limited treatment options.

In summary, we describe a novel mechanism by which alcohol causes failure of the hepatic firewall, by changing hepatic macrophage composition and phenotype, thus contributing to reduced CRIg expression in the liver. Reduced CRIg results in decreased removal of translocating pathobionts, which might not only contribute to more severe liver disease, but also to an increased rate of systemic infections in patients with end-stage liver disease (cirrhosis) or alcoholic hepatitis. CRIg is therefore an attractive target for treatment of alcohol-associated liver diseases.

## Methods

### Human study

Liver explants from six patients with severe alcoholic hepatitis, who underwent liver transplantation, and three liver tissues from donors (control) were obtained from Clinical Resource for Alcoholic Hepatitis Investigations (NIH R24 AA025017) at Johns Hopkins University (Baltimore, MD). Tissues were excised from explanted livers in patients with severe alcoholic hepatitis during liver transplantation, or wedge biopsies from the donor livers. The protocol was approved by the Johns Hopkins Medicine Institutional Review Boards (IRB00107893) and patients were enrolled after written informed consent was obtained.

### Mice

C57BL/6 mice were purchased from Charles River and used in Fig. 5 and Supplementary Fig. 2a. All mice used in other figures were bred in the animal facility at University of California, San Diego (La Jolla, CA). *CRIg*^*−/−*^ mice on a C57BL/6 background have been described (Genentech)^23,27^). Heterozygous female (+/−) and wild-type (y/+) or knock-out (y/−) male mice were used for breeding. *CRIg*^*−/−*^/*Tlr2*^*−/−*^ mice on a C57BL/6 background were generated by crossing *CRIg*^*−/−*^ mice with *Tlr2*^*−/−*^ mice.

Male mice (age, 9–12 weeks) were placed on a chronic–binge ethanol diet (NIAAA model) as described^17^. Mice were fed with the Lieber–DeCarli diet and the caloric intake from ethanol was 0% on days 1–5 and 36% from day 6 until the end of the study period. At day 16, mice were gavaged with a single dose of ethanol (5 g/kg body weight) in the early morning and sacrificed 9 h later. Pair-fed control mice received a diet with an isocaloric substitution of dextrose.

Female mice (age, 9–12 weeks) were placed on Lieber DeCarli diet for 8 weeks as previously described^34^. The caloric intake from ethanol was 0% on day 1, 10% on days 2 and 3, 20% on days 4 and 5, 30% from day 6 until the end of 6 weeks, and 36% for the last 2 weeks. On the last day, mice were gavaged with a single dose of ethanol (5 g/kg body weight) in the early morning and sacrificed 9 h later. Pair-fed control mice received a diet with an isocaloric substitution of dextrose.

To study the effect of *E. faecalis* on *CRIg*^*−/−*^ and *CRIg*^*−/−*^/*Tlr2*^*−/−*^ mice, *E. faecalis* (5 × 10^8^ colony forming units (CFUs)) were gavaged every third day, starting from day 6 through day 15 of ethanol feeding (see above). Administration every third day was necessary, given that *E. faecalis* does not colonize mice^17^.

In studies using CRIg-Ig, wild-type male mice were placed on a chronic–binge ethanol feeding model. CRIg-Ig or control-Ig (12 mg/kg BW) were injected intravenously 1 day before ethanol binge. CRIg-Ig was obtained from Genentech and was generated by fusing the extracellular domain of CRIg to the Fc portion of murine IgG1^23^. Murine anti-gp120 IgG1 (Bio X Cell) was used as control Ig as described^23,27^.

Mice were housed with a light cycle of 12 h of light (6 a.m.–6 p.m.) and a dark cycle of 12 h of darkness (6 p.m.–6 a.m.) at a temperature between 20 and 22 °C and a relative humidity between 40% and 70%. All animal studies were reviewed and approved by the Institutional Animal Care and Use Committee of the University of California, San Diego. The studies were performed in accordance with all relevant ethical regulations regarding the use of research animals.

### RNA sequencing

Previously published high-throughput transcriptome profiling by RNA sequencing with liver samples was used as described^28^. 41 patients with different phenotypes were selected: 12 patients with early alcoholic steatohepatitis, 11 patients with non-severe alcoholic hepatitis (with Maddrey discriminant function (MDF ≤ 32), and 18 patients with severe alcoholic hepatitis (MDF > 32). Controls (*n* = 10) were non-diseased livers obtained from biopsied single nodules, whose histology was without significant alterations. Transcripts were quantified using RSEM package^41^. Data was normalized and expressed as Transcripts Per Kilobase Million or TPM. The raw and phenotypic data are available through the Database of Genotypes And Phenotypes (dbGAP) of the National Center for Biotechnology Information (NCBI) under the Study Accession Id phs001807.v1.p1.

### Single cell RNA sequencing analysis

Previously published scRNAseq data following QC and annotation^30^ was downloaded from a publicly available server^42^. All analysis was performed in the Seurat R package (version 3.2.3). Data was initially subsetted only for cells from the 5 healthy and 2 alcohol-related cirrhotic livers (patient IDs cirrhotic 2 and cirrhotic 3)^30^. Cells from these patients which were annotated as mononuclear phagocytes (MP) based on the original published analyses were isolated for re-analysis, using the built-in functions of Seurat to normalize the cells per 10,000 transcripts and then log(*x* + 1) transformed allowing the resulting values to be scaled. Finally, the uniform Approximation and Projection Method (UMAP) was used to construct a data visualization graph using 1 to 10 principal components as determined by the dataset variability shown in principal component analysis (PCA). For this analysis, the genes of interest included “*CD68*”, “*TIMD4*”, “*CD5L*”, “*MARCO*”, “*CRIg*”, “*CD9*”, “*TREM2*” and “*S100A12*”. To identify how the expression of these genes differ between cells previously annotated as tissue monocytes (TMo), scar-associated macrophages (SAMac) and Kupffer cells (KC) in healthy and alcohol-related liver disease liver tissue, UMAP visualizations, violin plots and dot plots were generated using Seurat functions in conjunction with the ggplot2 package.

### Immunofluorescence staining

Liver sections were embedded in OCT compound. 5 μm frozen sections were then cut. To determine CRIg expression levels in controls and patients with alcoholic hepatitis, liver sections were stained with anti-huCRIg (1:500) (Genentech) and anti-CD68 (1:200) (Agilent Dako) primary antibodies. To determine CRIg expression levels in control- and ethanol-fed mice, liver sections were stained with anti-muCRIg (1:5,000) (Genentech) and anti-F4/80 (1:50) (Biolegend) primary antibodies. To determine T cell and neutrophil infiltration in all groups of ethanol-fed mice, liver sections were stained with anti-CD3e (1:300) (Biolegend) and anti-Ly6G (1:200) (Biolegend) primary antibodies. Representative pictures from both groups are shown. Five high-power fields of each sample were randomly selected for quantification using ImageJ and Qupath^43^.

### Real-time quantitative PCR

RNA was extracted from mouse tissues and cDNA was generated^17^. Primer sequences for mouse genes were obtained from the NIH qPrimerDepot. All primers used in this study are listed in Supplementary Table 1. Gene expression was determined with Sybr Green (Bio-Rad Laboratories) using ABI StepOnePlus real-time PCR system. The qPCR value was normalized to 18S.

### Bacterial culture

To make GFP producing *E. faecalis*, 1 μg of the plasmid pBSU101^44^ was transformed into a cytolytic *E. faecalis* strain^35^. All *E. faecalis* strains were grown statically in brain heart infusion (BHI) broth or on BHI agar plate at 37 °C. 125 μg/ml spectinomycin was added when GFP-produced *E. faecalis* strain was grown.

### Kupffer cell isolation and cell culture experiments

Primary mouse Kupffer cells were isolated by two-step collagenase-pronase perfusion followed by three-layer discontinuous density gradient centrifugation with 8.2% (w/v) and 14.5% (w/v) Nycodenz (Accurate Chemical and Scientific Corporation) to obtain Kupffer-cell fraction. 5 × 10^5^ Kupffer cells were plated on six-well plates and cultured with RPMI 1640 medium (Fisher Scientific) containing 10% (v/v) FBS. RNA was extracted and used for qPCR. Cell culture medium was replaced to be RPMI 1640 without serum, before the stimulation started. Kupffer cells from *CRIg*^*−/−*^ mice and their wild-type littermates were isolated and treated with PBS as vehicle, LPS (ENZO Life Sciences; 10 ng/ml), heat-killed *E. faecalis* (MOI 10:1), TNF (R&D Systems; 2 ng/ml), or IL1B (R&D Systems; 2 ng/ml) for 8 h. To check whether CRIg-bound *E. faecalis* can activate pathogen recognition receptor signaling, CRIg-Ig (100 μg/ml) or control-Ig (100 μg/ml) was incubated with 10^5^ *E. faecalis* at room temperature for 2 h and fixed by 1% paraformaldehyde. Bacteria were washed three times with PBS, and Kupffer cells were stimulated for 8 h.

To determine cell viability, Kupffer cells from *CRIg*^*−/−*^ mice and their wild-type littermates were isolated and plated on 96-well plates, cultured with RPMI 1640 medium (Fisher Scientific) containing 10% (v/v) FBS overnight. Fresh medium was then changed, and lactate dehydrogenase was measured 4 h following medium change in the supernatant (Fisher Scientific).

### Confocal microscopy

*CRIg*^*−/−*^ mice and their wild-type littermates were subjected to the chronic–binge ethanol feeding model. One hour after ethanol-binge, GFP-*E. faecalis* (2 × 10^7^ CFUs) was injected intravenously. Animals were sacrificed 1 h after bacterial injection and hepatic Kupffer cells were isolated. Cells were then stained with APC anti-F4/80 antibody (BioLegend) and sorted using anti-APC microbeads (Miltenyi Biotec). Before microscopy, cells were put on poly-l-lysine-coated slides. Pictures were taken with ×630 magnification. For quantification, five high-power fields of each sample were randomly selected and the number of internalized GFP-*E. faecalis* was counted. The phagocytic index was then calculated.

### Bacterial load

To determine bacterial load from different tissues, mice were placed on the chronic–binge ethanol diet. One hour after the ethanol binge, *E. faecalis* (5 × 10^9^ CFUs) were gavaged to each mouse. Animals were sacrificed 20 min after bacteria gavage and tissues were harvested. Each tissue was weighed and resuspended into 500 μl PBS. 100 μl of each homogenized tissue sample was then plated onto a BHI agar plate with 125 μg/ml spectinomycin; the plates were then incubated at 37 °C overnight. Colony numbers of each sample were then counted and CFUs were calculated.

### Flow cytometry of CRIg binding to bacteria

CRIg-Ig protein was labeled with Alexa Fluor 647 using a protein labeling kit (Fisher Scientific). Labeled CRIg-Ig was incubated with *E. faecalis* (2 × 10^5^ CFUs) or *E. coli* (2 × 10^5^ CFUs) at room temperature for 2 h. Bacteria were washed three times with PBS, resuspended in 1% paraformaldehyde, fixed on ice for 30 min, and subjected to flow cytometry, using the software SpectroFlo 2.2.0.

### Biochemical analysis

Serum levels of ALT were determined using Infinity ALT kit (Thermo Scientific). Hepatic triglyceride levels were measured using Triglyceride Liquid Reagents kit (Pointe Scientific). Serum levels of ethanol were measured using Ethanol Assay kit (BioVision). Serum levels of cytokines were measured using Proinflammatory Panel 1 (mouse) kit (Meso Scale Diagnostics). All results were measured using the software SoftMax Pro 7.0.3.

### Staining procedures

To determine lipid accumulation, liver sections were embedded in OCT compound. 8 μm frozen sections were then cut and stained with Oil Red O (Sigma-Aldrich). To check liver fibrosis, formalin-fixed tissue samples were embedded in paraffin and stained with Sirius red. Five high-power fields were randomly selected for quantification.

### Statistical analysis

Results are expressed as mean ± s.e.m. (except when stated otherwise). Significance of two groups or multiple groups were evaluated using two-sided unpaired Student’s *t*-test, two-sided unpaired Mann–Whitney test, one-way analysis of variance (ANOVA) with Tukey’s post-hoc test, Kruskal–Wallis test with Dunn’s post-hoc test, or two-way ANOVA, respectively. Exact *P* values for all comparisons, together with group size for each group, were listed in Supplementary Data 1. All results were generated from at least two independent technical replicates. Statistical analyses were performed using GraphPad Prism v7.04. A *P* < 0.05 was considered to be statistically significant (adjusted for multiple comparison when performing multiple tests).

### Reporting summary

Further information on research design is available in the Nature Research Reporting Summary linked to this article.

## Supplementary information

Supplementary Information

Description of Additional Supplementary Files

Supplementary Dataset 1

Reporting Summary

## Data Availability

The data that support the findings of this study are available from the author (B.S.) upon reasonable request. Source data are provided as a Source Data file. Original raw single-cell RNA-seq data have been deposited in the Gene Expression Omnibus (GEO) under accession GSE136103 and processed/annotated data available from https://datashare.ed.ac.uk/handle/10283/3433^42^. Original raw and phenotypic data from liver RNA sequencing are available through the Database of Genotypes And Phenotypes (dbGAP) of the National Center for Biotechnology Information (NCBI) under the Study Accession ID phs001807.v1.p1^28^. Source data are provided with this paper.

## Source data

Source Data

## References

[1] Lozano R (2012). Global and regional mortality from 235 causes of death for 20 age groups in 1990 and 2010: a systematic analysis for the Global Burden of Disease Study 2010. Lancet.

[2] Rehm J, Samokhvalov AV, Shield KD (2013). Global burden of alcoholic liver diseases. J. Hepatol..

[3] Rehm J (2014). Burden of disease associated with alcohol use disorders in the United States. Alcohol.: Clin. Exp. Res..

[4] Lee BP, Vittinghoff E, Dodge JL, Cullaro G, Terrault NA (2019). National trends and long-term outcomes of liver transplant for alcohol-associated liver disease in the United States. JAMA Intern. Med..

[5] Maddrey WC (1978). Corticosteroid therapy of alcoholic hepatitis. Gastroenterology.

[6] Mathurin P (2003). Early change in bilirubin levels is an important prognostic factor in severe alcoholic hepatitis treated with prednisolone. Hepatology.

[7] Forrest EH (2005). Analysis of factors predictive of mortality in alcoholic hepatitis and derivation and validation of the Glasgow alcoholic hepatitis score. Gut.

[8] Dominguez M (2008). A new scoring system for prognostic stratification of patients with alcoholic hepatitis. Am. J. Gastroenterol..

[9] Lucey MR, Mathurin P, Morgan TR (2009). Alcoholic hepatitis. N. Engl. J. Med..

[10] Mathurin P, Lucey MR (2012). Management of alcoholic hepatitis. J. Hepatol..

[11] Thursz MR (2015). Prednisolone or pentoxifylline for alcoholic hepatitis. N. Engl. J. Med..

[12] Gao B (2020). Functional microbiomics reveals alterations of the gut microbiome and host co-metabolism in patients with alcoholic hepatitis. Hepatol. Commun..

[13] Jiang L (2020). Intestinal virome in patients with alcoholic hepatitis. Hepatology.

[14] Lang S (2020). Intestinal fungal dysbiosis and systemic immune response to fungi in patients with alcoholic hepatitis. Hepatology.

[15] Lang S (2020). Changes in the fecal bacterial microbiota associated with disease severity in alcoholic hepatitis patients. Gut Microbes.

[16] Chu H (2020). The *Candida albicans* exotoxin candidalysin promotes alcohol-associated liver disease. J. Hepatol..

[17] Duan Y (2019). Bacteriophage targeting of gut bacterium attenuates alcoholic liver disease. Nature.

[18] Zhong W (2020). Paneth cell dysfunction mediates alcohol-related steatohepatitis through promoting bacterial translocation in mice: role of zinc deficiency. Hepatology.

[19] Stärkel P, Schnabl B (2016). Bidirectional communication between liver and gut during alcoholic liver disease. Semin. Liver Dis..

[20] Lang S, Schnabl B (2020). Microbiota and fatty liver disease—the known, the unknown, and the future. Cell Host Microbe.

[21] Balmer ML (2014). The liver may act as a firewall mediating mutualism between the host and its gut commensal microbiota. Sci. Transl. Med..

[22] Flannagan RS, Jaumouillé V, Grinstein S (2012). The cell biology of phagocytosis. Annu. Rev. Pathol..

[23] Helmy KY (2006). CRIg: a macrophage complement receptor required for phagocytosis of circulating pathogens. Cell.

[24] Wiesmann C (2006). Structure of C3b in complex with CRIg gives insights into regulation of complement activation. Nature.

[25] Zeng Z (2016). CRIg functions as a macrophage pattern recognition receptor to directly bind and capture blood-borne Gram-positive bacteria. Cell Host Microbe.

[26] Katschke KJ (2007). A novel inhibitor of the alternative pathway of complement reverses inflammation and bone destruction in experimental arthritis. J. Exp. Med..

[27] Yuan X, Yang B-H, Dong Y, Yamamura A, Fu W (2017). CRIg, a tissue-resident macrophage specific immune checkpoint molecule, promotes immunological tolerance in NOD mice, via a dual role in effector and regulatory T cells. Elife.

[28] Argemi J (2019). Defective HNF4alpha-dependent gene expression as a driver of hepatocellular failure in alcoholic hepatitis. Nat. Commun..

[29] MacParland SA (2018). Single cell RNA sequencing of human liver reveals distinct intrahepatic macrophage populations. Nat. Commun..

[30] Ramachandran P (2019). Resolving the fibrotic niche of human liver cirrhosis at single-cell level. Nature.

[31] Bertola A, Mathews S, Ki SH, Wang H, Gao B (2013). Mouse model of chronic and binge ethanol feeding (the NIAAA model). Nat. Protoc..

[32] Moresco EMY, LaVine D, Beutler B (2011). Toll-like receptors. Curr. Biol..

[33] Yimin (2013). Contribution of toll-like receptor 2 to the innate response against *Staphylococcus aureus* infection in mice. PLoS ONE.

[34] Chu H (2021). The selective PPAR-delta agonist seladelpar reduces ethanol-induced liver disease by restoring gut barrier function and bile acid homeostasis in mice. Transl. Res.

[35] Llorente C (2017). Gastric acid suppression promotes alcoholic liver disease by inducing overgrowth of intestinal *Enterococcus*. Nat. Commun..

[36] Fu W, Wojtkiewicz G, Weissleder R, Benoist C, Mathis D (2012). Early window of diabetes determinism in NOD mice, dependent on the complement receptor CRIg, identified by noninvasive imaging. Nat. Immunol..

[37] Vogt L (2006). VSIG4, a B7 family-related protein, is a negative regulator of T cell activation. J. Clin. Investig..

[38] Jung K (2012). Protective role of V-set and immunoglobulin domain-containing 4 expressed on kupffer cells during immune-mediated liver injury by inducing tolerance of liver T- and natural killer T-cells. Hepatology.

[39] van Lookeren Campagne M, Verschoor A (2018). Pathogen clearance and immune adherence “revisited”: immuno-regulatory roles for CRIg. Semin. Immunol..

[40] Li J (2017). VSIG4 inhibits proinflammatory macrophage activation by reprogramming mitochondrial pyruvate metabolism. Nat. Commun..

[41] Li B, Dewey CN (2011). RSEM: accurate transcript quantification from RNA-Seq data with or without a reference genome. BMC Bioinforma..

[42] Ramachandran, P., Henderson, N. & Wilson-Kanamori, J. R. Resolving the fibrotic niche of human liver cirrhosis at single-cell level - Seurat RData object. (University of Edinburgh Centre for Inflammation Research, 2019). 10.1038/s41586-019-1631-3 PMC6876711 31597160

[43] Bankhead P (2017). QuPath: open source software for digital pathology image analysis. Sci. Rep..

[44] Aymanns S, Mauerer S, van Zandbergen G, Wolz C, Spellerberg B (2011). High-level fluorescence labeling of Gram-positive pathogens. PLoS ONE.

