# Supplementary material for: CRIg on liver macrophages clears pathobionts and protects against alcoholic liver disease
Source: Nat Commun. 2021 Dec 9;12:7172. doi: 10.1038/s41467-021-27385-3 (PMC8660815; doi:10.1038/s41467-021-27385-3)
Supplement: Supplementary file 1 — Supplementary Information [file 41467_2021_27385_MOESM1_ESM.pdf]

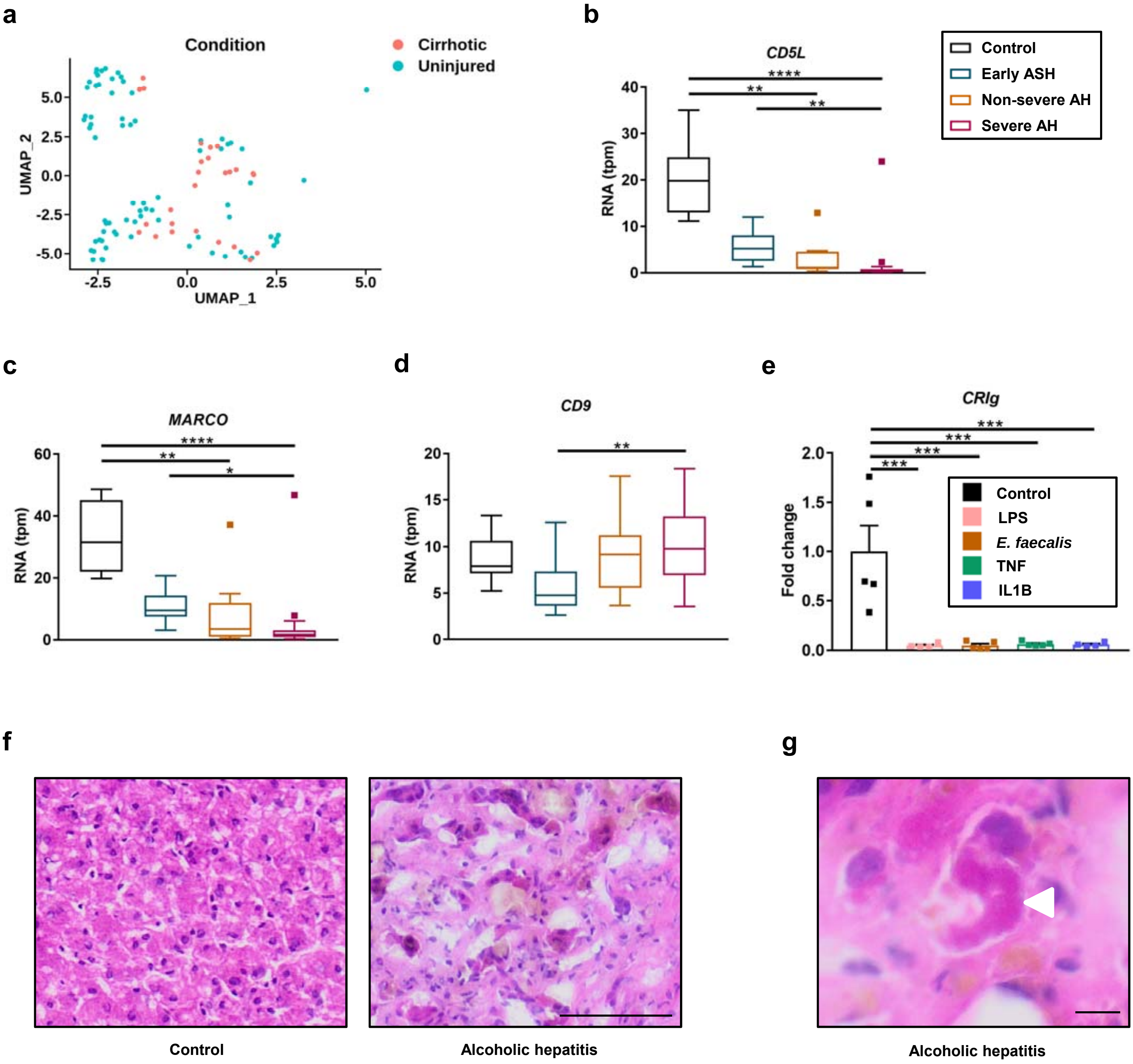

Supplementary Figure 1. Alcohol-related cirrhotic patients have different liver macrophage composition.

(a) UMAP annotating whether macrophages in scRNAseq analysis derive from patients with healthy or alcohol-related cirrhotic livers. (b–d) Hepatic levels of *CD5L, MARCO* and *CD9* from RNA sequencing data of human liver tissues from subjects without liver disease (Control; n=10), patients with early alcoholic steatohepatitis (Early ASH; n=12), non-severe alcoholic hepatitis (Non-severe AH; n=11) and severe alcoholic hepatitis (Severe AH; n=18). (e) Primary mouse Kupffer cells were treated with LPS (10 ng/ml), heat-killed *E. faecalis* (MOI 10:1), TNF (2 ng/ml), IL1B (2 ng/ml), or PBS (vehicle) for 8 hours. mRNA levels of *CRIg*. (f and g) Representative H&E-stained liver sections. White arrow: Mallory hyaline. Scale bar=100 μm (f) or 10μm (g). Results are expressed as median with interquartile range (b–d) or mean ± s.e.m (e). *P* value is determined by two-sided Kruskal-Wallis test with Dunn's post-hoc test (b–d), or One-way ANOVA with Tukey´s post-hoc test (e). **P*<0.05, ***P*<0.01, ****P*<0.001, *****P*<0.0001

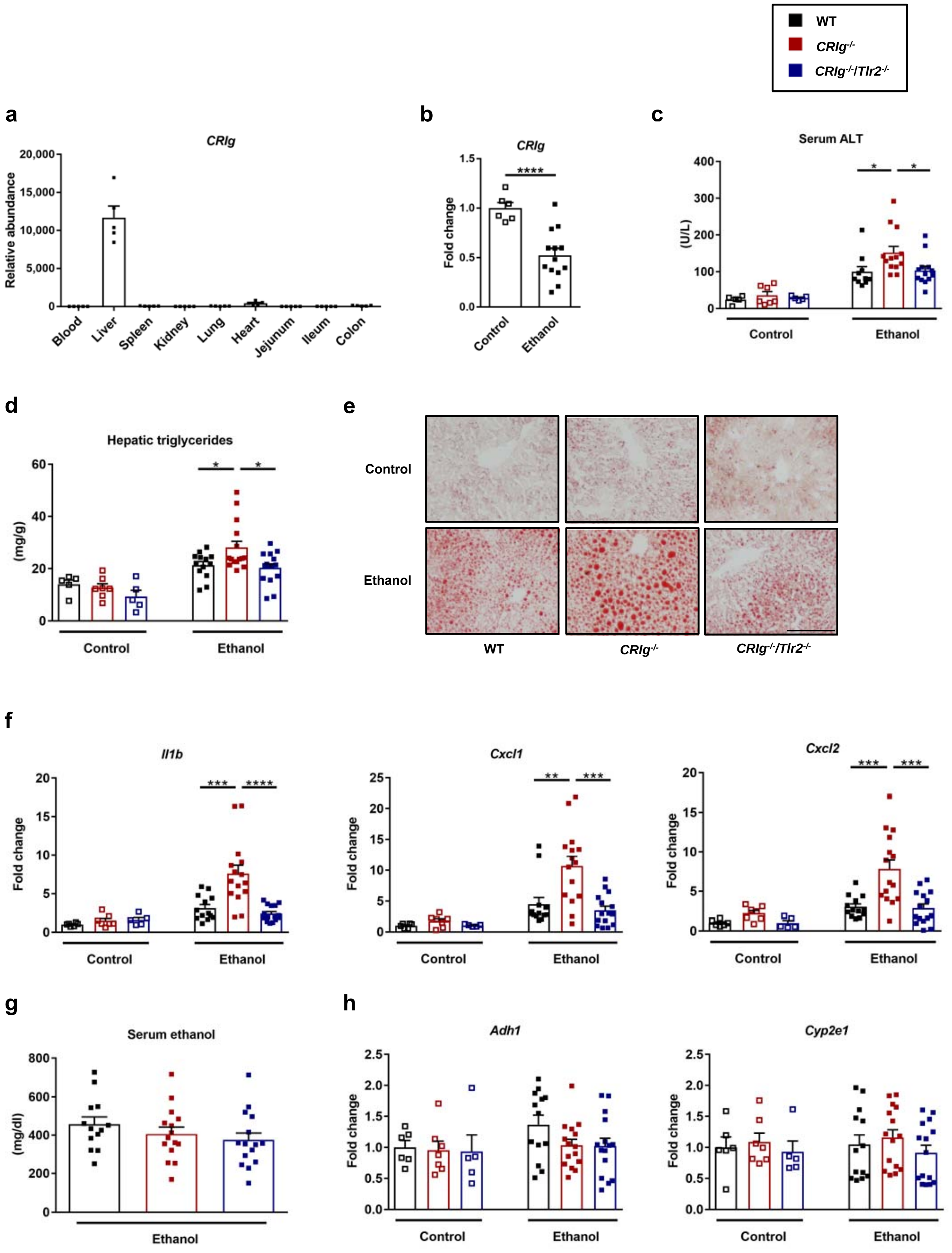

WT
CRIg-/-
CRIg-/-/Tlr2-/-
a
CRIg
Relative abundance
20,000
15,000
10,000
5,000
0
Blood
Liver
Spleen
Kidney
Lung
Heart
Jejunum
Ileum
Colon
b
CRIg
Fold change
1.5
1.0
0.5
0.0
****
Control
Ethanol
c
Serum ALT
(U/L)
400
300
200
100
0
*
*
Control
Ethanol
d
Hepatic triglycerides
(mg/g)
60
40
20
0
*
*
Control
Ethanol
e
Control
Ethanol
WT
CRIg-/-
CRIg-/-/Tlr2-/-
f
Il1b
Fold change
20
15
10
5
0
***
****
Control
Ethanol
Cxcl1
Fold change
25
20
15
10
5
0
**
***
Control
Ethanol
Cxcl2
Fold change
20
15
10
5
0
***
***
Control
Ethanol
g
Serum ethanol
(mg/dl)
800
600
400
200
0
Ethanol
h
Adh1
Fold change
2.5
2.0
1.5
1.0
0.5
0.0
Control
Ethanol
Cyp2e1
Fold change
2.5
2.0
1.5
1.0
0.5
0.0
Control
Ethanol

Supplementary Figure 2. *CRIg$^{-/-}$* mice have more severe ethanol-induced liver damage. (a) RNA was extracted from different tissues in WT mice and cDNA was generated. mRNA levels of *CRIg* were then determined by qPCR. (b–h) WT, *CRIg$^{-/-}$*, and *CRIg$^{-/-}$/Tlr2$^{-/-}$* mice were placed on the chronic–binge ethanol diet. (b) Hepatic level of mRNA encoding CRIg in WT mice. (c) Serum levels of ALT. (d) Hepatic triglyceride content. (e) Representative oil red O-stained liver sections. (f) Hepatic levels of mRNAs encoding inflammatory cytokines and chemokines IL1B, CXCL1, and CXCL2. (g) Serum levels of ethanol in ethanol-fed mice. (h) Hepatic levels of *Adh1* and *Cyp2e1* mRNAs. Scale bar=100 μm. Results are expressed as mean ± s.e.m (a–d, f–h). *P* values are determined by two-sided Student's t-test (b) and One-way ANOVA with Tukey´s post-hoc test (c, d, f–h). *$P<0.05$, **$P<0.01$, ***$P<0.001$, ****$P<0.0001$.

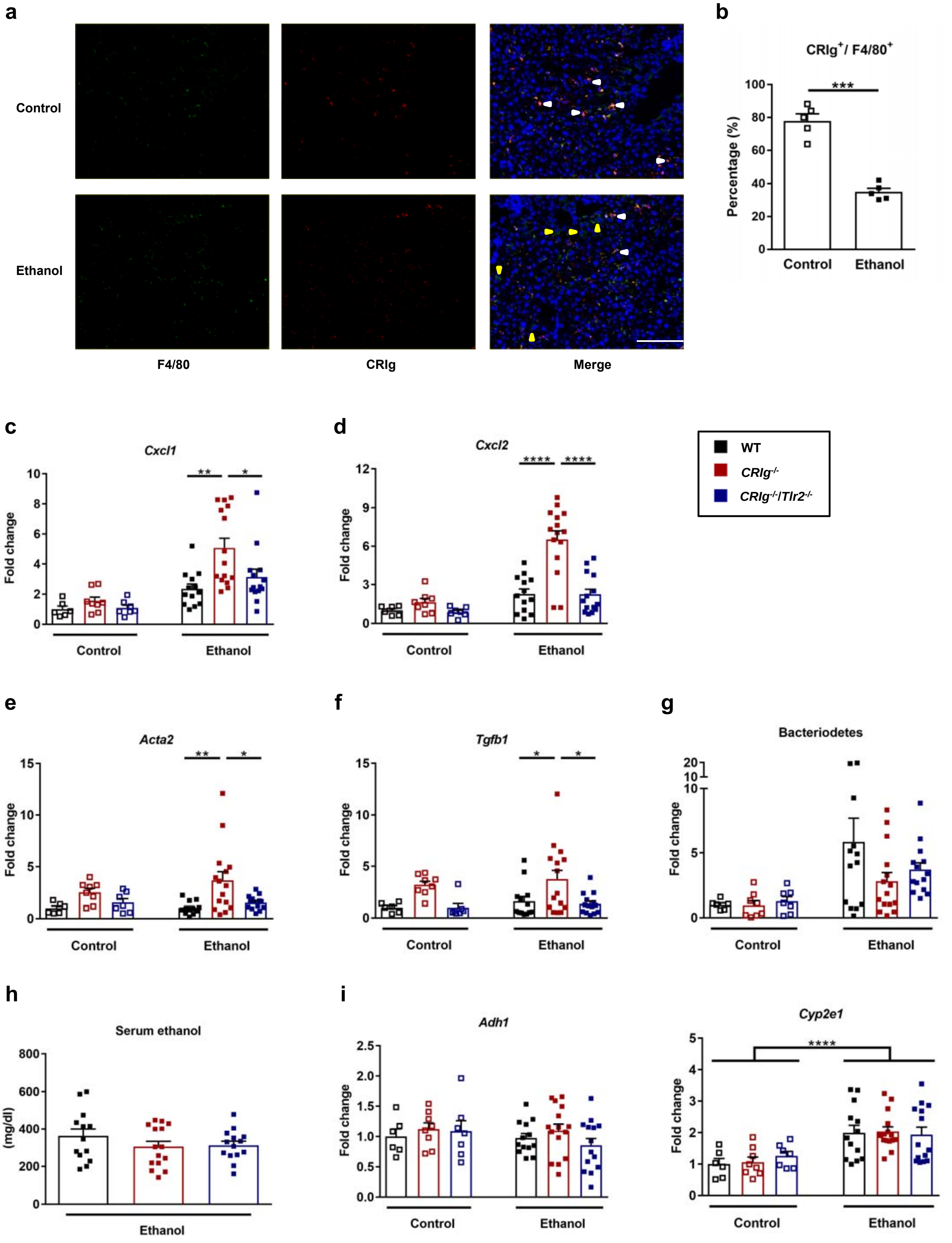

a
Control
Ethanol
F4/80
CRIg
Merge
b
CRIg+/ F4/80+
Percentage (%)
Control
Ethanol
c
Cxcl1
d
Cxcl2
WT
CRIg-/-
CRIg-/-/Tlr2-/-
e
Acta2
f
Tgfb1
g
Bacteriodetes
Fold change
h
Serum ethanol
(mg/dl)
i
Adh1
Cyp2e1

Supplementary Figure 3. Deletion of CRIg does not affect absorption and hepatic metabolism of ethanol.

WT, *CRIg*$^{-/-}$, and *CRIg*$^{-/-}$/*Tlr2*$^{-/-}$ mice were placed on the Lieber DeCarli ethanol diet model for 8 weeks. (a) Representative liver sections of F4/80 and CRIg immunofluorescence staining in WT mice. White arrow: macrophages expressing both F4/80 and CRIg; Yellow arrow: macrophages expressing F4/80 only. (b) Quantification of the stained liver sections. (c–f) Hepatic levels of mRNAs encoding *Cxcl1, Cxcl2, Acta2*, and *Tgfb1*. (g) Hepatic DNA levels of Bacteriodetes, normalized to total amount of bacteria using universal 16S primers. (h, i) Serum levels of ethanol and hepatic levels of *Adh1* and *Cyp2e1* mRNAs. Scale bar=100 μm. Results are expressed as mean ± s.e.m. *P* values are determined by two-sided Student's t-test (b) and one-way ANOVA with Tukey´s post-hoc test (c–i). *P* value for the difference between the mice on the control vs the ethanol diet fed mice determined by two-way ANOVA (h). **P*<0.05, ***P*<0.01, ****P*<0.001, *****P*<0.0001.

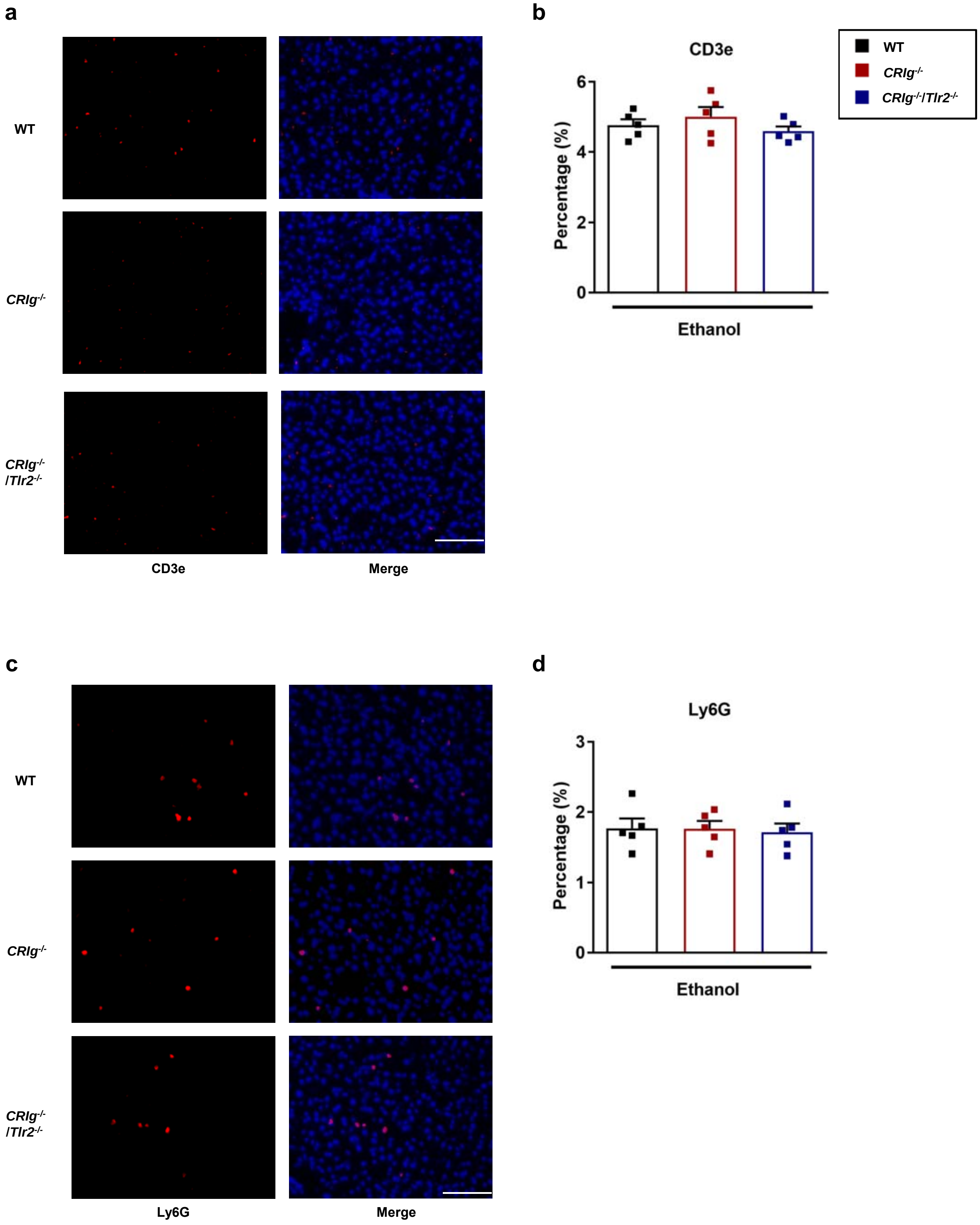
a
WT
CRIg-/-
CRIg-/-
/Tlr2-/-
CD3e
Merge
b
CD3e
WT
CRIg-/-
CRIg-/-/Tlr2-/-
Percentage (%)
6
4
2
0
Ethanol
c
WT
CRIg-/-
CRIg-/-
/Tlr2-/-
Ly6G
Merge
d
Ly6G
Percentage (%)
3
2
1
0
Ethanol

Supplementary Figure 4. Hepatic T cell and neutrophil infiltration following chronic ethanol administration.

WT, *CRIg*$^{-/-}$, and *CRIg*$^{-/-}$/*Tlr2*$^{-/-}$ mice were placed on the Lieber DeCarli ethanol diet model for 8 weeks. (a) Representative liver sections of CD3e staining showing T cells. (b) Quantification of the stained liver sections. (c) Representative liver sections of Ly6G staining showing neutrophils. (d) Quantification of the stained liver sections. Scale bar=100 μm. Results are expressed as mean ± s.e.m (b and d). *P* values are determined by One-way ANOVA with Tukey´s post-hoc test (b and d).

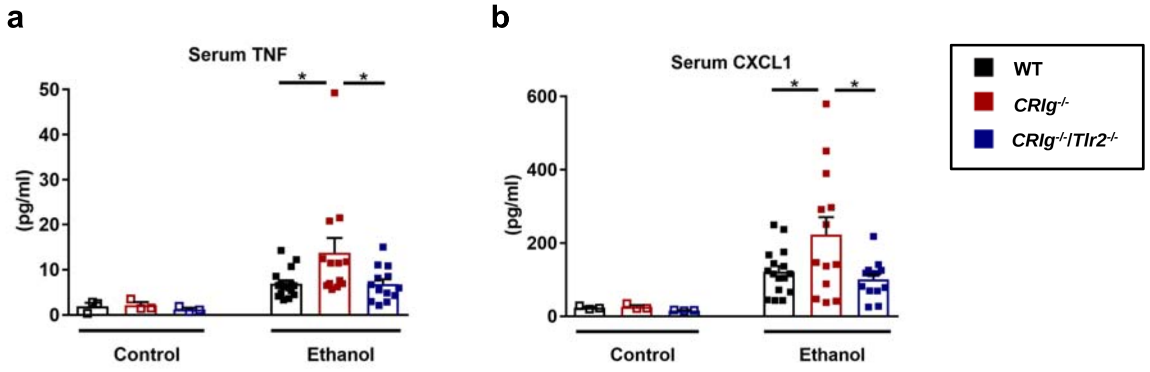

Supplementary Figure 5. ***CRIg**$^{-/-}$* **mice have increased systemic inflammation.**
WT, *CRIg*$^{-/-}$ and *CRIg*$^{-/-}$/*Tlr2*$^{-/-}$ mice were placed on the chronic–binge ethanol diet and gavaged with a cytolytic *E. faecalis* strain ($5x10^8$ colony forming units (CFUs)) every third day. (a–b) Serum levels of TNF and CXCL1. Results are expressed as mean ± s.e.m. *P* values among groups of mice fed with control diet or ethanol diet are determined by One-way ANOVA with Tukey´s post-hoc test. *P<0.05.

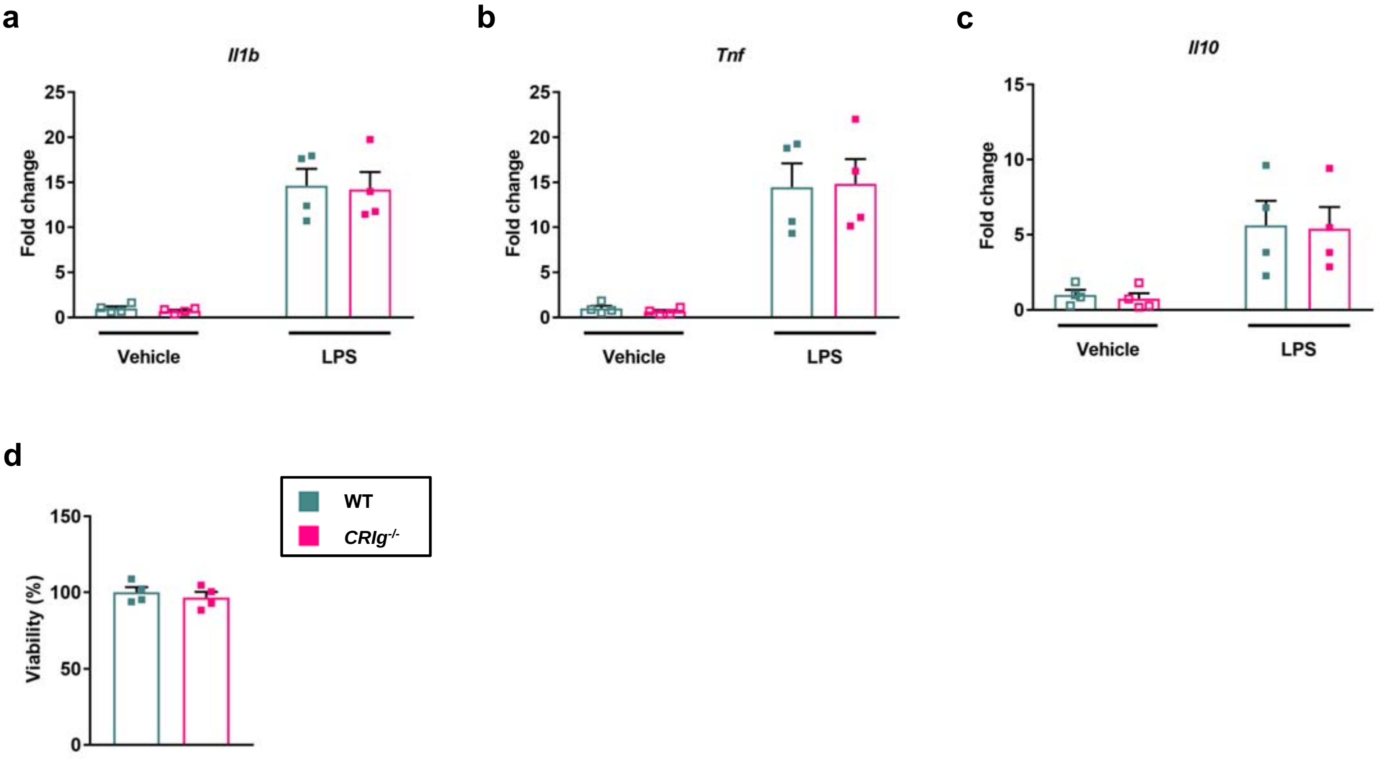

Supplementary Figure 6. *CRIg*-deficient Kupffer cells have similar phenotype as compared with wild-type Kupffer cells.
Kupffer cells were isolated from *CRIg*$^{-/-}$ mice and their wild-type littermates. (a–c) Cells were treated with LPS (10 ng/ml), or PBS (vehicle). mRNA levels of cytokines *Il1b*, *Tnf* and *Il10*. (d) Cell viability was measured by lactate dehydrogenase in the supernatant. The mean survival rate of wild-type cells was set as 100%. Results are expressed as mean ± s.e.m. *P* values are determined by two-sided Student's t-test.

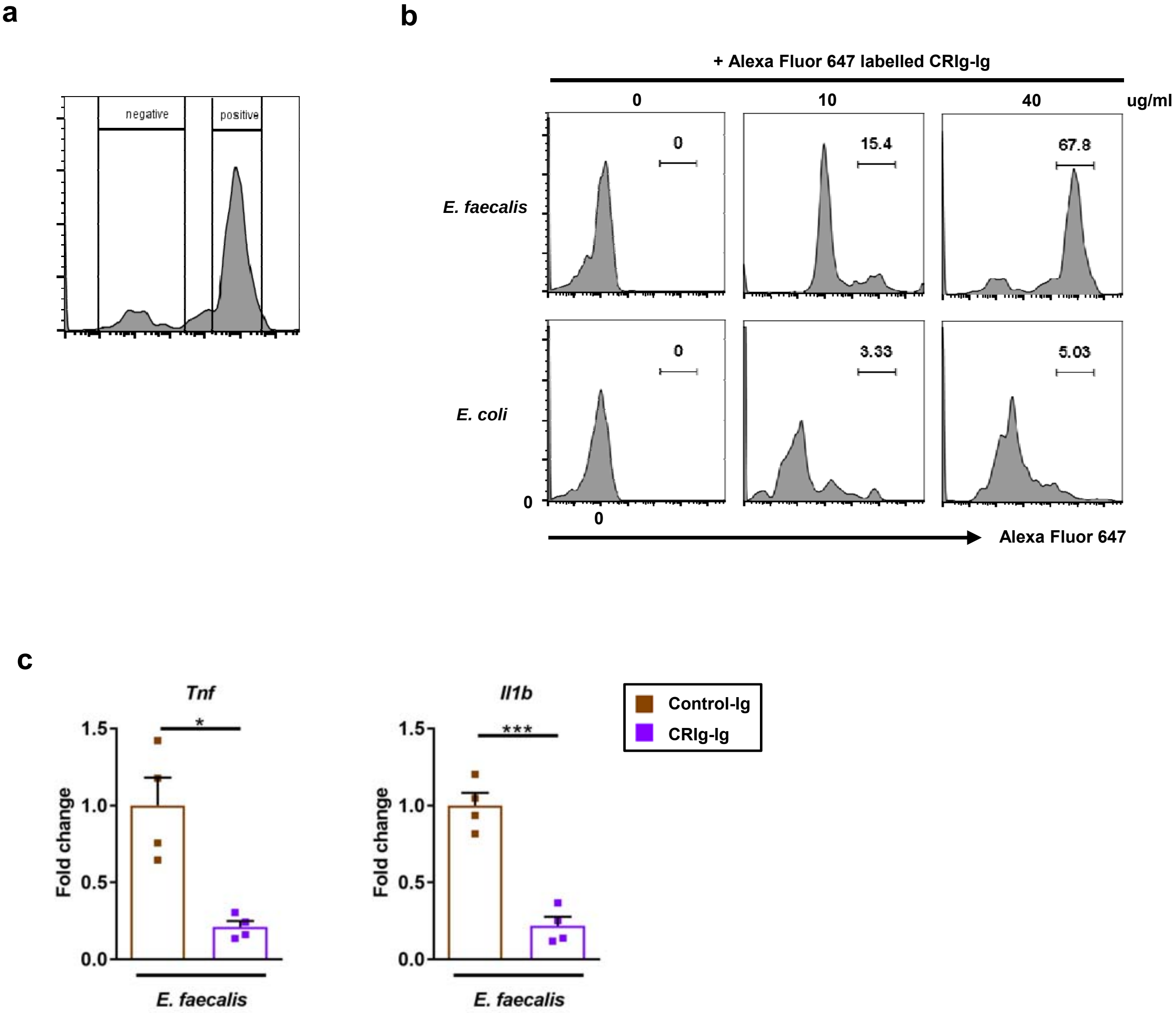

Supplementary Figure 7. CRIg-Ig binds to *E. faecalis* and prevents induction of inflammatory gene expression.

(a) Gating strategy for flow cytometry. (b) *E. faecalis* or *E. coli* were incubated with indicated concentrations of Alexa Fluor 647 labelled CRIg-Ig. Binding was detected by flow cytometry. (c) Control-Ig (100 µg/ml) or CRIg-Ig (100 µg/ml) was incubated with *E. faecalis* (10^5 CFUs) at room temperature for 2 hours, fixed and then added to primary isolated mouse Kupffer cells for 8 hours. mRNA levels of inflammatory cytokines *Tnf* and *Il1b* were assessed. Results are expressed as mean ± s.e.m (c). *P* values determined by two-sided Student's t-test (c). **P*<0.05, ****P*<0.001

Supplementary Table 1: Primers used in this study.

Gene	Primer	Nucleotide Sequence
Mouse *18S*	F	5'-AGTCCCTGCCCTTTGTACACA-3'
	R	5'-CGATCCCAGGGCCTCACTA-3'
Mouse *Acta2*	F	5'-GTTCAGTGGTGCCTCTGTCA-3'
	R	5'-ACTGGGACGACATGGAAAAG-3'
Mouse *Adh1*	F	5'-GGGTTCTCAACTGGCTATGG-3'
	R	5'-ACAGACAGACCGACACCTCC-3'
Mouse *Col1a1*	F	5'-TAGGCCATTGTGTATGCAGC-3'
	R	5'-ACATGTTCAGCTTTGTGGACC-3'
Mouse *CRIg*	F	5'-CCTGGGCCACCTAATAGTGC-3'
	R	5-'TGTAGCCTCTCAGGGGATCAT-3'
Mouse *Cxcl1*	F	5'-TGCACCCAAACCGAAGTC-3'
	R	5'-GTCAGAAGCCAGCGTTCACC-3'
Mouse *Cxcl2*	F	5'-AAAGTTTGCCTTGACCCTGAA-3'
	R	5'-CTCAGACAGCGAGGCACATC-3'
Mouse *Cyp2e1*	F	5'-CTTAGGGAAAACCTCCGCAC-3'
	R	5'-GGGACATTCCTGTGTTCCAG-3'
Mouse *Il1b*	F	5'-GGTCAAAGGTTTGGAAGCAG-3'
	R	5'-TGTGAAATGCCACCTTTTGA-3'
Mouse *TGFb1*	F	5'-GGAGAGCCCTGGATACCAAC-3'
	R	5'-CAACCCAGGTCCTTCCTAAA-3'
Universal bacterial *16S*	F	5'-GTGSTGCAYGGYTGTCGTCA-3'
	R	5'-ACGTCRTCCMCACCTTCCTC-3'
Bacteriodetes *16S*	F	5'-GGCGACCGGCGCACGGG-3'
	R	5'-GRCCTTCCTCTCAGAACCC-3'
Firmicutes *16S*	F	5'-GGAGYATGTGGTTTAATTCGAAGCA-3'
	R	5'-AGCTGACGACAACCATGCAC-3'
Gammaproteobacteria *16S*	F	5'-TCGTCAGCTCGTGTYGTGA-3'
	R	5'-CGTAAGGGCCATGATG-3'
