# Supplementary material for: CRIg on liver macrophages clears pathobionts and protects against alcoholic liver disease
Source: Nat Commun. 2021 Dec 9;12:7172. doi: 10.1038/s41467-021-27385-3 (PMC8660815; doi:10.1038/s41467-021-27385-3)
Supplement: Supplementary file 2 — Description of Additional Supplementary Files [file 41467_2021_27385_MOESM2_ESM.pdf]

File name: Supplementary Data 1

Description: For each figure and supplementary figure, exact *P* values for all comparisons and group size for each group were listed.
