# Supplementary material for: CRIg on liver macrophages clears pathobionts and protects against alcoholic liver disease
Source: Nat Commun. 2021 Dec 9;12:7172. doi: 10.1038/s41467-021-27385-3 (PMC8660815; doi:10.1038/s41467-021-27385-3)
Supplement: Supplementary file 4 — Reporting Summary [file 41467_2021_27385_MOESM4_ESM.pdf]

nature research

Corresponding author(s): BERND SCHNABL

Last updated by author(s): Oct 3, 2021

Reporting Summary

Nature Research wishes to improve the reproducibility of the work that we publish. This form provides structure for consistency and transparency in reporting. For further information on Nature Research policies, see our Editorial Policies and the Editorial Policy Checklist.

Statistics

For all statistical analyses, confirm that the following items are present in the figure legend, table legend, main text, or Methods section.

n/a	Confirmed	
☐	☒	The exact sample size (n) for each experimental group/condition, given as a discrete number and unit of measurement
☐	☒	A statement on whether measurements were taken from distinct samples or whether the same sample was measured repeatedly
☐	☒	The statistical test(s) used AND whether they are one- or two-sided<br>*Only common tests should be described solely by name; describe more complex techniques in the Methods section.*
☐	☒	A description of all covariates tested
☐	☒	A description of any assumptions or corrections, such as tests of normality and adjustment for multiple comparisons
☐	☒	A full description of the statistical parameters including central tendency (e.g. means) or other basic estimates (e.g. regression coefficient) AND variation (e.g. standard deviation) or associated estimates of uncertainty (e.g. confidence intervals)
☐	☒	For null hypothesis testing, the test statistic (e.g. F, t, r) with confidence intervals, effect sizes, degrees of freedom and P value noted<br>*Give P values as exact values whenever suitable.*
☒	☐	For Bayesian analysis, information on the choice of priors and Markov chain Monte Carlo settings
☒	☐	For hierarchical and complex designs, identification of the appropriate level for tests and full reporting of outcomes
☒	☐	Estimates of effect sizes (e.g. Cohen's d, Pearson's r), indicating how they were calculated

Our web collection on statistics for biologists contains articles on many of the points above.

Software and code

Policy information about availability of computer code

Data collection	All biochemical assays were measured using SoftMax Pro 7.0.3; qPCRs were run with StepOnePlus real-time PCR system; Liver histological pictures were taken with DP Controller and DP Manager (Olympus). All pictures were viewed using ImageJ and QuPath.
Data analysis	Staining:<br>NIH ImageJ 1.50i<br>QuPath<br>Statistical analyses:<br>GraphPad Prism v7.04<br>Flow cytometry:<br>SpectroFlo 2.2.0

For manuscripts utilizing custom algorithms or software that are central to the research but not yet described in published literature, software must be made available to editors and reviewers. We strongly encourage code deposition in a community repository (e.g. GitHub). See the Nature Research guidelines for submitting code & software for further information.

Data

Policy information about availability of data

All manuscripts must include a data availability statement. This statement should provide the following information, where applicable:

- Accession codes, unique identifiers, or web links for publicly available datasets
- A list of figures that have associated raw data
- A description of any restrictions on data availability

The data that support the findings of this study are available from the authors upon reasonable request. Source data are provided with this paper. Original raw

single-cell RNA-seq data have been deposited in the Gene Expression Omnibus (GEO) under accession GSE136103 and processed/annotated data available from https://datashare.ed.ac.uk/handle/10283/3433. Original raw and phenotypic data from liver RNA sequencing are available through the Database of Genotypes And Phenotypes (dbGAP) of the National Center for Biotechnology Information (NCBI) under the Study Accession Id phs001807.v1.p1

Field-specific reporting

Please select the one below that is the best fit for your research. If you are not sure, read the appropriate sections before making your selection.

☒ Life sciences ☐ Behavioural & social sciences ☐ Ecological, evolutionary & environmental sciences

For a reference copy of the document with all sections, see nature.com/documents/nr-reporting-summary-flat.pdf

Life sciences study design

All studies must disclose on these points even when the disclosure is negative.

Sample size	No power analyses or other calculations were used to predetermine sample sizes. Sample sizes were chosen based on prior literature using similar experimental paradigms (Nature Communications. 2017;8:2137; Nature. 2019;575:505-1511)
Data exclusions	No data were excluded
Replication	More than two technical replicates (from different cohorts, on different dates), as well as biological replicates were performed to ensure data reproducibility. All replications were successful.
Randomization	Mice of similar age and weight were randomly assigned to experimental and control groups. Primary isolated mouse Kupffer cells were randomly assigned to experimental and control groups.
Blinding	We had no specific methods to blind the investigators during the experiments, but all mice were treated equally at the same time. Each round of technical replicate contains all groups.

Reporting for specific materials, systems and methods

We require information from authors about some types of materials, experimental systems and methods used in many studies. Here, indicate whether each material, system or method listed is relevant to your study. If you are not sure if a list item applies to your research, read the appropriate section before selecting a response.

Materials & experimental systems

n/a	Involved in the study	
☐	☒	Antibodies
☒	☐	Eukaryotic cell lines
☒	☐	Palaeontology and archaeology
☐	☒	Animals and other organisms
☐	☒	Human research participants
☒	☐	Clinical data
☒	☐	Dual use research of concern

Methods

n/a	Involved in the study	
☒	☐	ChIP-seq
☐	☒	Flow cytometry
☒	☐	MRI-based neuroimaging

Antibodies

Antibodies used	Mouse anti-human CRIg, clone 3D10 (Genentech)<br>Mouse anti-human CD68, clone PG-M1 (Agilent Dako, Cat#IS61330-2)<br>Rat anti-mouse F4/80, APC, clone BM8 (Biolegend, Cat#123116)<br>Mouse anti-mouse CRIg, clone 14G8 (Genentech)<br>Hamster anti-mouse CD3e, clone 145-2C11 (Biolegend, Cat#100301)<br>Rat anti-mouse Ly6G, clone 1A8 (Biolegend, Cat#127601)
Validation	Mouse anti-human CRIg and anti-mouse CRIg antibodies were gifts from M. van Lookeren Campagne from Genentech and were generated in Helmy et al. 2006; Mouse anti-human CD68 antibody has been used in Chen et al. 2015; Rat anti-mouse F4/80 antibody has been used in Ying et al. 2019; Hamster anti-mouse CD3e antibody has been used in Lin et al. 2017; Rat anti-mouse Ly6G antibody has been used in Maybin et al. 2018;

Animals and other organisms

Policy information about studies involving animals; ARRIVE guidelines recommended for reporting animal research

Laboratory animals	Female and male C57BL/6 mice (age, 9–12 weeks) (strain: wild type, CRIg-/-, CRIg-/-/Tlr2-/-)
Wild animals	No wild animals were involved in the study.
Field-collected samples	No field-collected samples were involved in the study
Ethics oversight	All animal studies were reviewed and approved by the Institutional Animal Care and Use Committee of the University of California, San Diego.

Note that full information on the approval of the study protocol must also be provided in the manuscript.

Human research participants

Policy information about studies involving human research participants

Population characteristics	Coded and de-identified liver explants from six patients with severe alcoholic hepatitis, who underwent liver transplantation, and three liver tissues from donors (control) were obtained from Clinical Resource for Alcoholic Hepatitis Investigations (NIH R24 AA025017) at Johns Hopkins University (Baltimore, MD). Tissues were excised from explanted livers in patients with severe alcoholic hepatitis during liver transplantation, or wedge biopsies from the donor livers.
Recruitment	Liver explants from six patients with severe alcoholic hepatitis, who underwent liver transplantation, and three liver tissues from donors (control) were used.
Ethics oversight	The protocol was approved by the Johns Hopkins Medicine Institutional Review Boards (IRB00107893) and patients were enrolled after written informed consent was obtained.

Note that full information on the approval of the study protocol must also be provided in the manuscript.

Flow Cytometry

Plots

Confirm that:

☒ The axis labels state the marker and fluorochrome used (e.g. CD4-FITC).

☒ The axis scales are clearly visible. Include numbers along axes only for bottom left plot of group (a 'group' is an analysis of identical markers).

☒ All plots are contour plots with outliers or pseudocolor plots.

☒ A numerical value for number of cells or percentage (with statistics) is provided.

Methodology

Sample preparation	CRIg-Ig protein was labelled with Alexa Fluor 647 using a protein labeling kit (Fisher Scientific). Labelled CRIg-Ig was incubated with E. faecalis (2 × 105 CFUs) or E. coli (2 × 105 CFUs) at room temperature for 2 hours. Bacteria were washed three times with PBS, resuspended in 1% paraformaldehyde, fixed on ice for 30 min, and subjected to flow cytometry.
Instrument	Cytek NL3000
Software	SpectroFlo v2.2.0
Cell population abundance	More than 10,000 cells were counted for each sample
Gating strategy	With the increasing concentration of the protein, more E. faecalis (positive) were fluorescently labelled, with obviously two subpolutions; while E. coli (negative) were not fluorescently labelled.

☒ Tick this box to confirm that a figure exemplifying the gating strategy is provided in the Supplementary Information.
